# Alternative- and focal therapy trends for prostate cancer: a total population analysis of in-patient treatments in Germany from 2006 to 2019

**DOI:** 10.1007/s00345-022-04024-0

**Published:** 2022-05-13

**Authors:** Luka Flegar, Aristeidis Zacharis, Cem Aksoy, Hendrik Heers, Marcus Derigs, Nicole Eisenmenger, Angelika Borkowetz, Christer Groeben, Johannes Huber

**Affiliations:** 1 Department of Urology, Philipps-University Marburg, Baldingerstr., 35043 Marburg, Germany; 2 Reimbursement Institute, Hürth, Germany; 3 Department of Urology, TU Dresden, Dresden, Germany

**Keywords:** Focal therapy, Prostate cancer, Population-based, Trends, HIFU, TOOKAD^®^, TULSA

## Abstract

**Purpose:**

Focal therapy (FT) offers an alternative approach for prostate cancer (PCa) treatment in selected patients. However, little is known on its actual establishment in health care reality.

**Patients and methods:**

We defined FT as high-intensity focused ultrasound (HIFU), hyperthermia ablation, cryotherapy, transurethral ultrasound ablation (TULSA) or vascular-targeted photodynamic therapy (VTP) TOOKAD^®^. We analyzed the nationwide German hospital billing database for a PCa diagnosis in combination with FT. For analyses on the hospital level, we used the reimbursement.INFO tool based on hospitals’ quality reports. The study period was 2006 to 2019.

**Results:**

We identified 23,677 cases of FT from 2006 to 2019. Considering all PCa cases with surgery, radiotherapy or FT, the share of FT was stable at 4%. The annual caseload of FT increased to a maximum of 2653 cases in 2008 (*p* < 0.001) and then decreased to 1182 cases in 2014 (*p* < 0.001). Since 2015, the cases of FT remained on a plateau around 1400 cases per year. The share of HIFU was stable at 92–96% from 2006 to 2017 and decreased thereafter to 75% in 2019 (*p* = 0.015). In 2019, VTP-TOOKAD^®^ increased to 11.5% and TULSA to 6%. In 2006, 21% (62/299) of urological departments performed FT and 20 departments reached > 20 FT procedures. In 2019, 16% (58/368) of urological departments performed FT and 7 departments reached > 20 FT. In 2019, 25 urological departments offered FT other than HIFU: 5 centers hyperthermia ablation, 11 centers VTP TOOKAD^®^, 3 centers cryotherapy, 6 centers TULSA.

**Conclusion:**

The FT development in Germany followed the Gartner hype cycle. While HIFU treatment is the most commonly performed FT, the share of newer FT modalities such as VTP-TOOKAD^®^ and TULSA is remarkably increasing.

**Supplementary Information:**

The online version contains supplementary material available at 10.1007/s00345-022-04024-0.

## Introduction

The concept of focal therapy (FT) for treatment of prostate cancer (PCa) has gained increasing importance worldwide in recent years offering an alternative to whole-gland treatment [1]. PCa is the most commonly diagnosed malignancy in the western world and represents the second leading cause of cancer-related death in men [2]. Standard treatment for localized PCa still consists of radical prostatectomy (RP) or radiation therapy (RT) in the majority of cases [3]. Alternatively, active surveillance (AS) can be offered for low-risk PCa. However, the observational approach of AS can impose a heavy burden on the patient [4]. Advances in imaging technology, biomarkers as well as improvements in prostate biopsy techniques result in diagnosing PCa at an earlier stage in general [5]. Therefore, a less radical and more precise treatment with fewer side effects can be offered as FT to selected patients with low- or intermediate-risk PCa. Various focal approaches are nowadays available [5, 6]. High-intensity focused ultra-sound (HIFU), cryotherapy, hyperthermia ablation, transurethral ultrasound ablation (TULSA), and vascular-targeted photodynamic therapy (VTP) with TOOKAD^®^ soluble are most commonly used. Clinical trials evaluating HIFU therapy for PCa started in the 1990s initially as whole-gland treatment and the focus of HIFU became more focal over time [7, 8]. First approaches in cryotherapy were described in the mid-1990s [9]. VTP with TOOKAD^®^ soluble has been approved by the European Medical Agency (EMA) in 2017 and has been clinically implemented in Germany since May 2018 [10]. TULSA is another new FT approach [11]. In general, all focal modalities are associated with lower side effects regarding incontinence and erectile dysfunction and provide reasonable short-term oncological safety [5, 12]. Reported Quality of life (QoL) after FT remains stable and decision-regret is low [13, 14]. Current guidelines recommend FT in the setting of clinical trials [15]. Critical pre-operative counseling and detailed post-operative follow-up with prostate biopsy at 12 months is required in patients undergoing FT [16].

Population-based studies that examined the utilization of FT for PCa are scarce. Therefore, our aim was to evaluate alternative- and focal therapy trends for PCa in Germany from 2006 to 2019.

## Patients and methods

We defined FT as HIFU, hyperthermia ablation, cryotherapy, TULSA or VTP TOOKAD^®^. The study period was 2006 to 2019.

### Database

We analyzed data from German hospitals’ quality reports and from the German Billing Database (Destatis). The German hospitals’ quality reports were used for identification of national providers while the Destatis database was used for analysis of all surgical procedures. We described the data extraction and cohort identification methods in previous studies [17, 18]. On an institutional level, we analyzed the annual FT caseload as well as the specific FT approaches with the reimbursement.INFO tool (Reimbursement Institute, Hürth, Germany) based on billing data from hospitals’ quality reports. We used the OPS code “5-602” representing FT. Further, we analyzed OPS code “5-602.1” (HIFU), “5-602.0” (hyperthermia ablation), “5-602.3” (cryotherapy), “5-602.5” (VTP), “5-601.a” (TULSA) and “5-602.y” (various). Map displays were created using the software “EasyMap 11.1 Standard Edition” (Lutum + Tappert DV-Beratung GmbH, Bonn, Germany).

The Destatis database collects reimbursement data of inpatient treatment since 2004. We included patients with the diagnosis of prostate cancer (ICD code “C61.0”) in combination with FT (OPS code “5-602”). We further analyzed specific FT approaches as described above in combination with the ICD code “C61.0”. To calculate the share of FT for PCa treatment, we used OPS code “5-604” representing radical prostatectomy (RP), OPS codes “8-520, 8-521, 8-522” representing radiation therapy (RT) as well as OPS codes “8-524 and 8-525” coding for brachytherapy.

### Statistical analysis

Data were presented by absolute and relative frequencies. To detect trends, over time linear regression models were implemented. We defined *p* < 0.05 to indicate statistical significance. We used SPSS 27.0 (IBM corp., Armonk, NY, USA) for our statistical analysis.

## Results

In total, we included 23,677 cases of FT between 2006 and 2019.

**Fig. 1:**
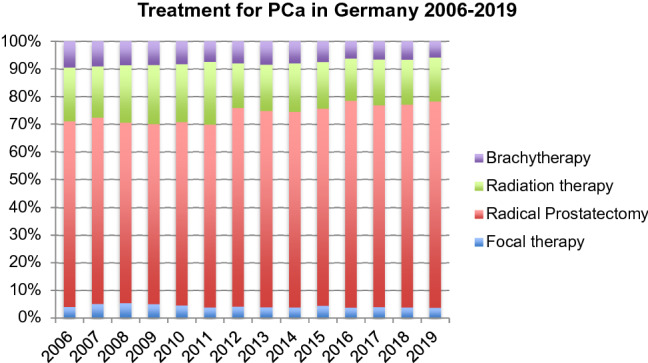
Overview of all major treatment options for PCa in % ( Source: Nationwide hospital billing database of the German Federal Statistical Office)

Figure 1 gives an overview of all major active treatment options for PCa. The share of FT within this group decreased slightly from 4.0% in 2006 to 3.8% in 2019 (*p* < 0.001). The share of RP increased from 67.0% in 2006 to 74.5% in 2019 (*p* = 0.004) while the share of RT decreased from 19.5% in 2006 to 15.9% in 2019 (*p* = 0.008). The share of brachytherapy decreased as well from 9.4% in 2006 to 5.8% in 2019 (*p* = 0.009).

**Fig. 2:**
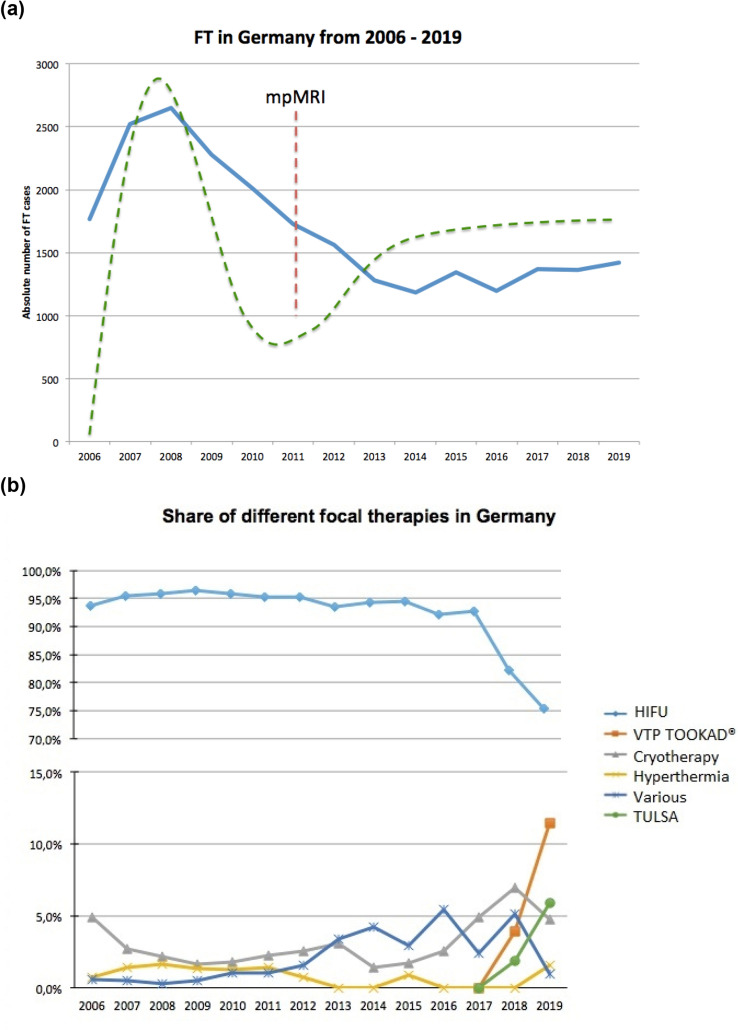
**a** Absolute numbers of FT from 2006 to 2019 (blue line) and the hype cycle (dashed green line). Red dashed line indicating start of use of mpMRI in clinical routine for PCa diagnostics and **b** share of different focal therapies from 2006 to 2019. ( Source: Nationwide hospital billing database of the German Federal Statistical Office)

Figure 2a presents the annual number of FT cases in Germany for patients with PCa, which increased initially by 50% from 1768 cases in 2006 to 2653 cases in 2008 (*p* < 0.001) and then decreased by 55% to 1182 cases in 2014 (*p* < 0.001). Since 2015, the cases of FT remained on a plateau around 1,400 cases per year. This development roughly follows the Gartner hype cycle for innovation and adoption of a new technology. However, the establishment of multiparametric magnetic resonance imaging (mpMRI) for PCa diagnostics since 2011 is likely to have mitigated the downturn.

Share of HIFU was stable over 90% from 2006 to 2017 and decreased to 75.3% in 2019 (*p* = 0.005) (Fig. 2b). Share of vascular-targeted photodynamic therapy increased from 4% in 2018 to 11.5% in 2019 (*p* = 0.041). The share for transurethral ultrasound ablation (TULSA) was 5.9% (*p* = 0.087) in 2019 and share of cryotherapy 4.7%, respectively (*p* = 0.147).

Relative to all performed FT, the proportion of patients > 70 years undergoing focal treatment for PCa increased from 57.1% in 2006 to 61.2% in 2019 (*p* = 0.029).

**Fig. 3:**
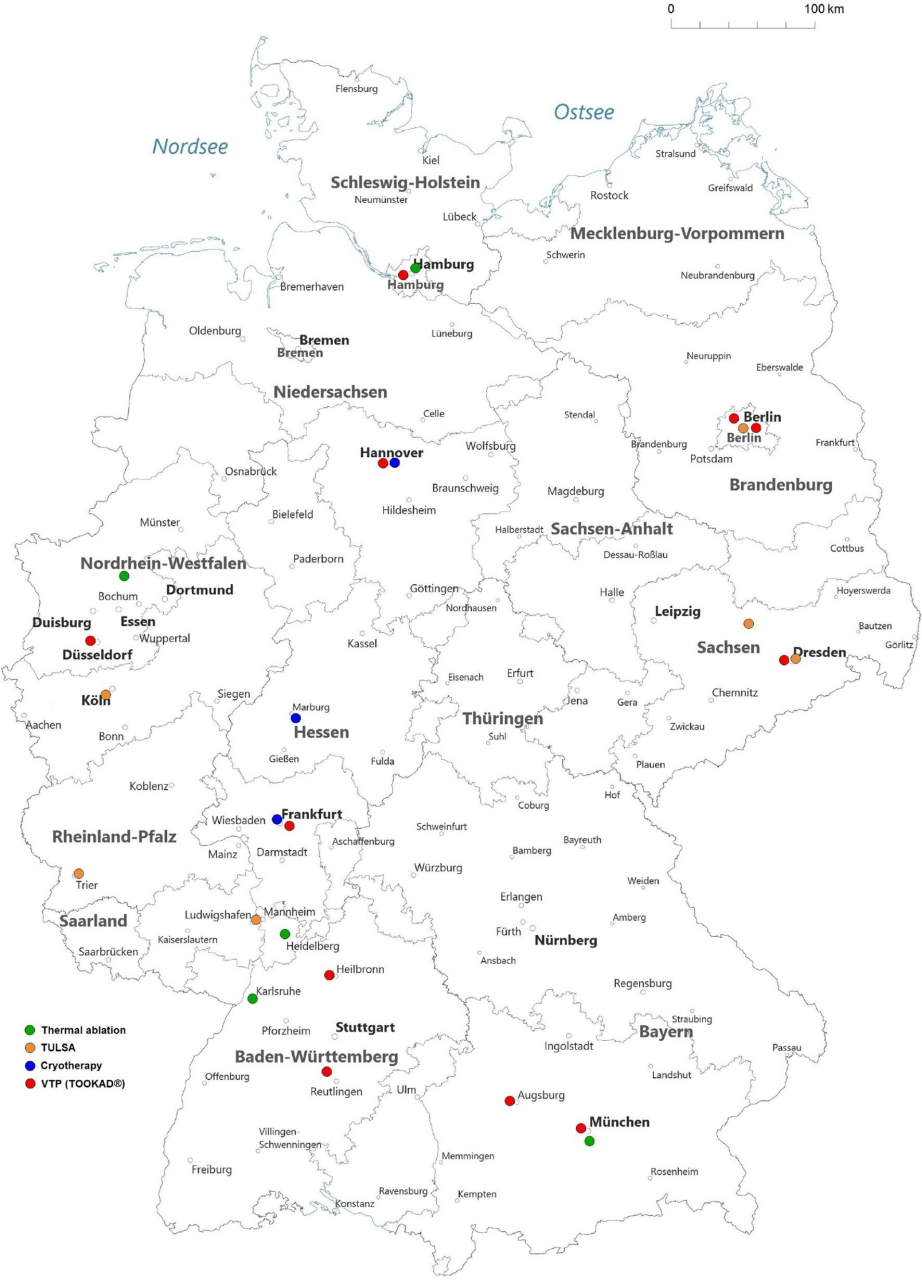
Overview of urologic departments offering thermal ablation, TULSA, cryotherapy and VTP TOOKAD^®^ for PCa treatment in Germany in 2019 ( Source: German hospitals’ quality reports)

In 2006, 5 of 41 urology departments (12%) performed more than 50 HIFU procedures per year, which decreased to 2 of 36 (6%) in 2019. In 2019, 3 departments (8%) performed 20–49 HIFUs and 31 (86%) departments performed < 20 HIFU procedures. Figure 3 (online supplement) gives an overview of the hospital caseload distribution for HIFU treatment in Germany in 2006 and 2019, respectively.

In 2019, 2 of 11 urology departments (18%) performed more than 10 VTP-TOOKAD^®^ procedures. In 2019, 5 departments (46%) performed 4–10 VTP-TOOKAD^®^ procedures and 4 (36%) performed < 4 VTP-TOOKAD^®^ procedures. Figure 4 (online supplement) shows all centers offering VTP TOOKAD^®^ throughout Germany in 2019.

In 2006, 21% (62/299) of urological departments performed FT and 20 departments reached > 20 FT procedures. In 2019, 16% (58/368) of urological departments performed FT and 7 departments reached > 20 FT. In 2019, 25 urologic departments offered FT other than HIFU: 5 centers hyperthermia ablation, 11 centers VTP TOOKAD^®^, 3 centers cryotherapy, 6 centers TULSA. Figure 3 gives an overview of various performed FTs in Germany in 2019.

## Discussion

Although FT offers an alternative treatment approach for selected patients with PCa for almost 3 decades, little is known on its actual establishment in health care reality. In the present study, we provided an analysis of recent population-based data showing that FT represents an established treatment option for PCa with a constant share of around 4% during the study period.

### Trends of FT for PCa treatment

Our longitudinal population-based study has several important findings. First, we observed that FT cases increased from 2006 to 2008 and then decreased with a low in 2014. Since 2015, cases of all focal modalities are on a plateau. We identified that the FT development in Germany followed the Gartner hype cycle for innovation and adoption of a new technology. Several studies described a similar trend for the implementation of a new medical service or product [19]. The hype cycle is characterized by five stages: technology trigger, peak of inflated expectations, trough of disillusionment, slope of enlightenment and plateau of productivity. The yearly case numbers of FT for PCa treatment during our study period followed this pattern. First focal approaches for PCa treatment were mentioned in the 1990s using HIFU and cryotherapy [7, 9]. In 2004, Onik introduced “male lumpectomy” using cryotherapy [20]. Since then, several new FT modalities have been invented and found their way into clinical practice [6, 11]. Especially the development of mpMRI and technical improvements of prostate biopsy for PCa detection promoted further implementation of FT [5, 21]. Advantages of FT are excellent functional outcomes regarding potency, continence and QoL with acceptable oncologic control of PCa [22]. Therefore, FT provides an attractive alternative for selected men with low- and intermediate risk PCa. Our data showed that in 2006, 21% of urological departments in Germany performed FT and 20 departments reached > 20 FT procedures. In 2019, 16% of urological departments offered FT and 7 departments reached > 20 FT. Especially the risk for tumor recurrence or progression after FT, which is not neglectable, could be the main reason for decreasing case numbers besides the hype cycle. The confrontation with the actual individual clinical courses of one’s own patients typically reduces the initial euphoria. After FT patients require a strict follow-up with mpMRI and control biopsy [10]. Further, since the German S3 guidelines for PCa recommended FT only within clinical trials until the 2021 publication update, reimbursement by health insurance for some FT procedures might not have been covered [16]. Therefore, some patients might have turned to private clinics that offer the desired FT. This migration of patients from statutory funding or private health insurance to facilities outside this regulatory framework could also explain a degression of FT in certain time periods. While analyzing our created maps, we noticed, that in all major cities (e.g., Berlin, Hamburg, Munich, Frankfurt, Cologne) at least one form of FT was accessible to patients in 2019. Besides HIFU, only VTP-TOOKAD^®^ was found as an offered FT in all larger regions of Germany (North, East, South and West).

### HIFU treatment

Second, we saw that HIFU was the most performed focal approach for PCa patients with a share of over 90% from 2006 to 2017. However, HIFU treatment was initially also used as a whole-gland treatment and centers in Regensburg and Munich were very active in this regard in earlier times. In 2019, the share of HIFU decreased to 75.3% while newer focal approaches such as VTP increased. Since HIFU compromises more than 90% of all FT modalities until 2017, its decreasing use was the major driver for low FT numbers in 2014. Ultimately, FT consists of a group of techniques, each of which also follows the hype cycle. Therefore, the most plausible explanation for this phenomenon seems to be a cessation of the hype for HIFU, while a little later the hype for VTP and TULSA started. The different FT techniques are also subject to changing popularity. Hopstaken et al. recently published a systematic review on FT. They described that of the 72 studies reporting on FT, over a third evaluated HIFU [23]. This is in line with our results showing that 60% of urological departments offered HIFU in 2019. However, HIFU’s dominant role in focal PCa treatment seems to slowly decrease. We found, that in 2006, 12% of urology departments performed more than 50 HIFU procedures per year, which decreased to 5.5% in 2019. We assume that the application of newer FT is the main reason for this decreasing trend of HIFU and we were able to show that in 2019, 25 urologic departments offered FT other than HIFU.

### Vascular-targeted photodynamic therapy and other FT approaches

Third, there was remarkable increase in the share of VTP-TOOKAD^®^ from 4% in 2018 to 11.5% in 2019 (*p* = 0.041). In total, 11 urology departments performed VTP-TOOKAD^®^ procedures in 2019 and 2 centers accomplished over 10 procedures. VTP in combination with TOOKAD^®^ soluble is available since 2018 for PCa treatment in Germany [10]. Azzouzi et al. were able to show with the PCM 301 trial that VTP significantly reduces the incidence of aggressive PCa in follow-up examinations [24]. VTP-TOOKAD^®^ is reimbursed by German health insurances if patients meet the inclusion criteria according to EMA approval [10]. This could partly explain the increase of this FT approach. Further, we noticed a steep increase in TULSA procedures for PCa since 2018 while cryotherapy is slowly increasing since 2014. A recently published single center study, evaluating 52 patients after TULSA, demonstrated the safety and early oncological efficacy of the procedure [25]. Mercader et al. described a low general complication profile for cryotherapy [26]. In contrary, hyperthermia ablation, which was offered in five German urological departments in 2019, did not increase significantly in share in the last years.

### FT in the elderly

Our results showed that relative to all performed FT, the proportion of patients > 70 years undergoing focal treatment for PCa increased from 57.1% in 2006 to 61.2% in 2019. A retrospective feasibility study investigating HIFU in the elderly showed that FT was safe and the long-term cancer control was adequate [27]. Therefore, FT may offer an alternative approach for elder patients with PCa, which do not qualify for other active treatments.

### Limitations and strengths

This study with more than 23.677 cases is the first to provide population-based data on FT for localized PCa in Germany. The present findings must be interpreted within the context of the study design. First, as described previously, the Destatis database lacks clinical information such as Gleason Score or tumor size [28]. Therefore, an accurate statement about share of patients with low- or intermediate risk PCa cannot be given. Second, the quality of population-based data is always inferior to case files and study records [29]. Third, there is no code available within the Destatis database to filter for AS, which represents an alternative treatment option for patients with low-risk PCa. Unfortunately, the code for irreversible electroporation was not available before 2020 in the OPS coding system (OPS code “5-602.6”) and, therefore, we were not able to include this FT modality in the present study. Especially, irreversible electroporation and TULSA are frequently performed in an outpatient setting or privately paid. Therefore, we were not able to cover these cases in our analysis. Further, the quality reports may be subject to documentation errors since they are prepared by the hospitals during routine care. Concerning HIFU treatment, we were not able to differentiate between a focal approach or a whole-gland treatment since there is only one OPS code. Since 2016, a new generation of HIFU devices is available that increases the potential of HIFU as a FT. However, keeping these limitations in mind, we were able to evaluate FT trends for PCa in Germany over a period of 14 years.

### Conclusion

We saw that FT development in Germany followed the Gartner hype cycle. HIFU treatment is still the most commonly performed FT in Germany. However, its share is slowly decreasing and the popularity of newer FT modalities such as VTP-TOOKAD^®^ and TULSA increased remarkably during the last years of our study. Altogether, 25 urologic departments offered FT other than HIFU in 2019.

## Supplementary Information

Below is the link to the electronic supplementary material.

Supplementary file1 (DOCX 3646 kb)

## Abbreviations

EMA: European Medical Agency
FT: Focal therapy
HIFU: High-intensity focal ultrasound
mpMRI: Multiparametric magnetic resonance imaging
QoL: Quality of life
RP: Radical prostatectomy
RT: Radiation therapy
TULSA: Transurethral ultrasound ablation
VTP: Vascular-targeted photodynamic therapy

## Declarations

### Availability of data and materials (data transparency)

The datasets used and analyzed during the current study are available from the corresponding author on request.
