# Supplementary material for: Alternative- and focal therapy trends for prostate cancer: a total population analysis of in-patient treatments in Germany from 2006 to 2019
Source: World J Urol. 2022 May 13;40(7):1645–52. doi: 10.1007/s00345-022-04024-0 (PMC9236973; doi:10.1007/s00345-022-04024-0)
Supplement: Supplementary file 1 — Supplementary file1 (DOCX 3646 kb) [file 345_2022_4024_MOESM1_ESM.docx]

**Online supplement**


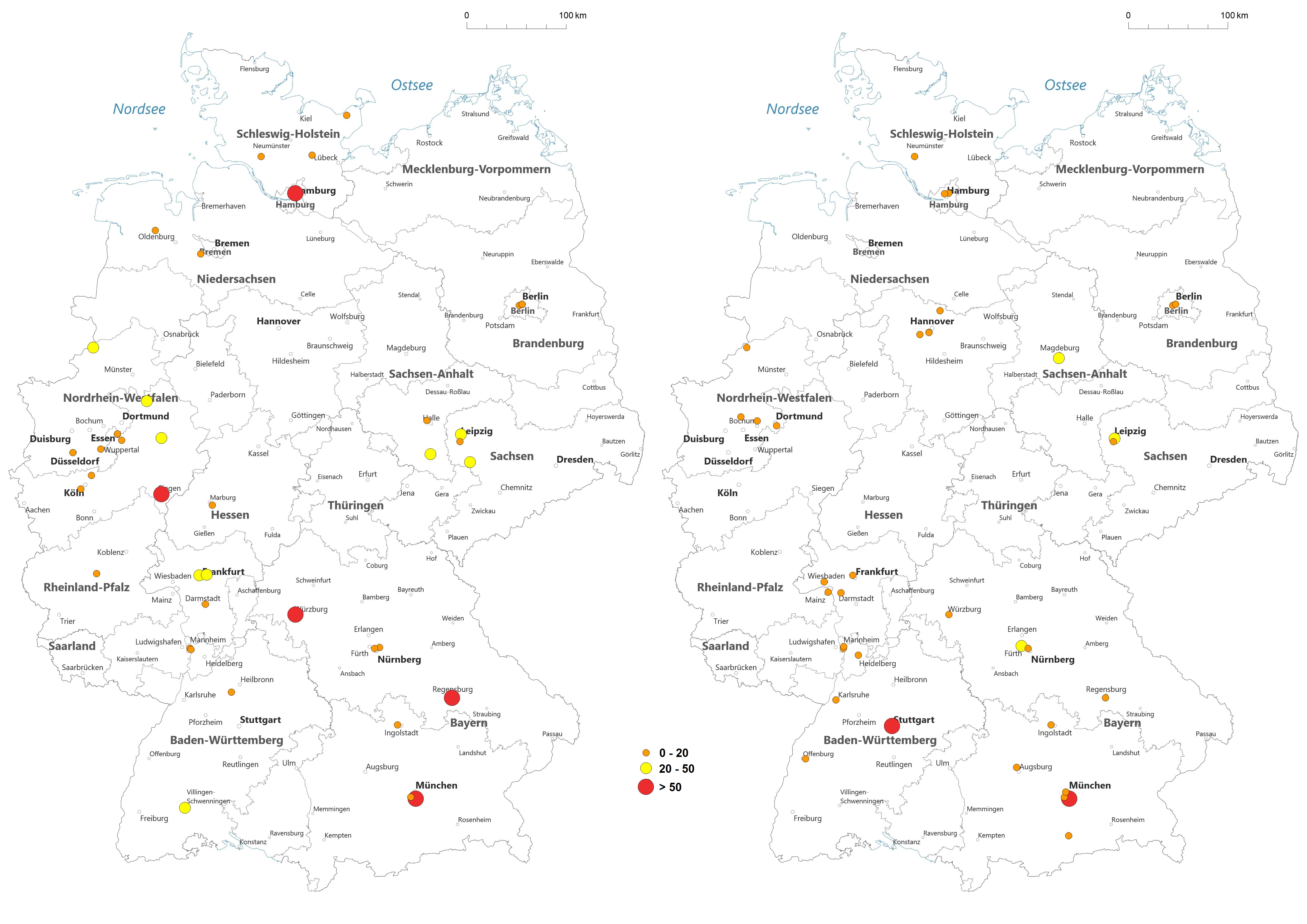


**Fig. 3.** Distribution of centers offering HIFU in 2006 (left) vs. 2019 (right) (caseload: 0-20; 20-50; >50 cases per year; Source: German hospitals’ quality reports).


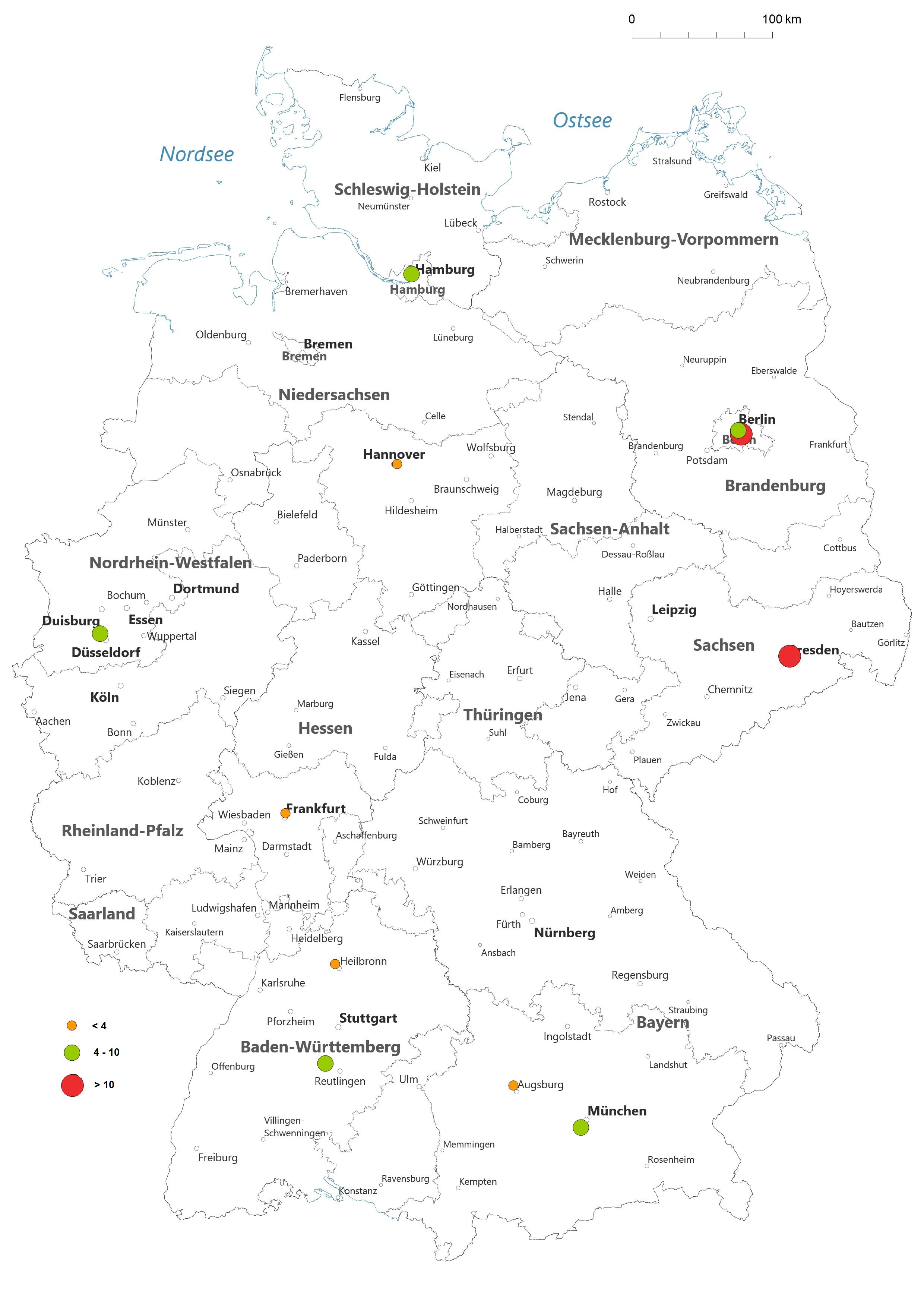


**Fig. 4.** Caseload distribution of centers offering VTP TOOKAD**®** in 2019 in Germany (caseload: 0-4; 4-10; >10 cases per year; Source: German hospitals’ quality reports).
